# Genetic dissection of 26 meat cut, meat quality and carcass traits in four pig populations

**DOI:** 10.1186/s12711-023-00817-y

**Published:** 2023-06-29

**Authors:** Lei Xie, Jiangtao Qin, Tianxiong Yao, Xi Tang, Dengshuai Cui, Liqing Chen, Lin Rao, Shijun Xiao, Zhiyan Zhang, Lusheng Huang

**Affiliations:** State Key Laboratory for Pig Genetic Improvement and Production Technology, Jiangxi Agricultural University, Nanchang, 330045 China

## Abstract

**Background:**

Currently, meat cut traits are integrated in pig breeding objectives to gain extra profit. However, little is known about the heritability of meat cut proportions (MCP) and their correlations with other traits. The aims of this study were to assess the heritability and genetic correlation of MCP with carcass and meat quality traits using single nucleotide polymorphism chips and conduct a genome-wide association study (GWAS) to identify candidate genes for MCP.

**Results:**

Seventeen MCP, 12 carcass, and seven meat quality traits were measured in 2012 pigs from four populations (Landrace; Yorkshire; Landrace and Yorkshire hybrid pigs; Duroc, and Landrace and Yorkshire hybrid pigs). Estimates of the heritability for MCP ranged from 0.10 to 0.55, with most estimates being moderate to high and highly consistent across populations. In the combined population, the heritability estimates for the proportions of scapula bone, loin, back fat, leg bones, and boneless picnic shoulder were 0.44 ± 0.04, 0.36 ± 0.04, 0.44 ± 0.04, 0.38 ± 0.04, and 0.39 ± 0.04, respectively. Proportion of middle cuts was genetically significantly positively correlated with intramuscular fat content and backfat depth. Proportion of ribs was genetically positively correlated with carcass oblique length and straight length (0.35 ± 0.08 to 0.45 ± 0.07) and negatively correlated with backfat depth (− 0.26 ± 0.10 to − 0.45 ± 0.10). However, weak or nonsignificant genetic correlations were observed between most MCP, indicating their independence. Twenty-eight quantitative trait loci (QTL) for MCP were detected by GWAS, and 24 new candidate genes related to MCP were identified, which are involved with growth, height, and skeletal development. Most importantly, we found that the development of the bones in different parts of the body may be regulated by different genes, among which *HMGA1* may be the strongest candidate gene affecting forelimb bone development. Moreover, as previously shown, *VRTN* is a causal gene affecting vertebra number, and *BMP2* may be the strongest candidate gene affecting hindlimb bone development.

**Conclusions:**

Our results indicate that breeding programs for MCP have the potential to enhance carcass composition by increasing the proportion of expensive cuts and decreasing the proportion of inexpensive cuts. Since MCP are post-slaughter traits, the QTL and candidate genes related to these traits can be used for marker-assisted and genomic selection.

**Supplementary Information:**

The online version contains supplementary material available at 10.1186/s12711-023-00817-y.

## Background

For a long time, the traditional pork sales model in China consisted in cutting the carcass into pieces following customers’ preferences [1, 2]. However, in recent years, many breeding firms have implemented centralized slaughtering, standardized quarantine, carcass cutting, and cold chain transportation to control the spread of African swine fever and other epidemic diseases. In addition, fast-paced and high-quality lifestyles and high-standard consumption concepts have established new market requirements [3]. As a result, precut and prepackaged cold meat is becoming increasingly popular. The prices of different cuts vary greatly, with up to two to three times differences between the highest and lowest price products. Notably, Qin [4] calculated the sum of sales of carcass cuts based on the average selling price of meat cuts and compared it with the total sale price of carcasses. He found that the sale of meat cuts represents at least 19% more economic revenue than the sale of carcasses, without considering costs such as labor and the depreciation of machines. As a result, breeders and scientists have become interested in investigating breeding programs to improve meat cut proportions (MCP) and in the genetic mechanisms underlying these traits.

As early as the 1970s, Whiteker et al. [5, 6] had already evaluated the weight and yield of different meat cuts. More recently, Overholt et al. [7] compared the differences in pork carcass cuts between barrows and gilts. Álvarez-Rodríguez and Teixeira [8] investigated the effect of slaughter weight and sex on carcass cuts based on three sequential target slaughter body weights of 17, 32, and 79 kg in Bisaro pigs and found that slaughter weight had a stronger impact on carcass cuts than sex. In our previous study, we observed that carcass weight, sex, and breed composition have significant effects on the weight and proportion of most meat cuts [9]. In addition, the coefficient of variation for MCP ranged from 4.2 to 25.3%, which indicates substantial heterogeneity between individuals [9]. Selecting elite pigs, including those with a higher proportion of high-price meat cuts, would benefit the pig industry. To implement a breeding program for improving MCP, it is necessary to first evaluate the genetic parameters and investigate the genetic architecture of these traits. Several studies have reported genetic parameters for meat cuts and found heritability estimates for carcass traits that were moderate to high [10–12]. However, these studies mainly focused on meat cuts under the standards of the United Nations Economic Commission for Europe (UNECE) and The Meat Buyer’s Guide from the North American Meat Processors Association (NAMP). In this study, the cut products followed the standards of Fresh and Frozen Pork and Pig By-Products (GB/T 9959) from the Standardization Administration of China, and included those reported in previous studies [10–12], as well as some novel meat cuts such as arm bones, scapula bone, chine bones, leg bones, and tail and pelvis bone. Between 2017 and 2018, the wholesale price for these bone meat cuts ranged from 11.5 to 17.7 yuan/kg, which is slightly lower than the wholesale price for the whole carcass (ranging from 14.5 to 19.4 yuan/kg), which is popular with consumers in China. Furthermore, the heritability estimates previously reported for meat cuts were mainly for commercial pigs, but estimates are also needed for purebred pigs.

In this study, we compared the heritability estimates for meat cuts between different breeds and crossbred populations and investigated their genetic correlations with carcass and meat quality traits, which will benefit decision-making in breeding programs for meat cut traits. Finally, we also performed a genome-wide association study (GWAS) to identify quantitative trait loci (QTL) for meat cut traits and, for the first time, the associated genes.

## Methods

### Ethics statement

All procedures involving animals followed the guidelines for the care and use of experimental animals approved by the State Council of the People’s Republic of China. The ethics committee of Jiangxi Agricultural University specifically approved this study.

### Animals and phenotype data

In total, 2012 pigs from Muyuan Food Co., Ltd. (Henan, China) were randomly sampled for the meat cut evaluation experiment, as described in Lei et al. [9]. In brief, four populations were used: 265 Landrace (LD, 95 sows and 170 barrows), 698 Yorkshire (YK, 435 sows and 263 barrows), 689 Landrace and Yorkshire hybrid (LY, 402 sows and 287 barrows), and 258 Duroc × Landrace × Yorkshire hybrid (DLY, 115 sows and 143 barrows). All pigs were raised under consistent feeding and nutritional conditions and were given the same commercial diets and access to water ad libitum*,* which were formulated to meet the recommendations of the Chinese National Feeding Standard (GB, 2004) at different growth phases. After 180 days of age, they were centrally slaughtered following standard pig slaughtering processes.

In the slaughterhouse, 12 carcass traits were measured, including carcass straight length (CSL), carcass oblique length (COL), thoracic length (THL), lumbar length (LUL), thoracic number (THN), lumbar number (LUN), single lumbar length (SLUL), shoulder backfat depth (SBD), 6th to 7th rib backfat depth (RBD), waist backfat depth (WBD), hip backfat depth (HBD), and mean of backfat depth (MBD). These traits were measured on the complete carcass, which was hung vertically by fixing the hind legs. Backfat depth refers to the thickness of the subcutaneous fat on the back of the carcass. After 24 h of acid excretion, the carcass was divided into three primal cuts (SC: shoulder cut, MC: middle cut, LC: leg cut), and 14 commercial meat cuts (BBS: boneless Boston shoulder, BPS: boneless picnic shoulder, FR: front ribs, AB: arm bones, SB: scapula bones, LO: loin, BE: belly, ribs, CB: chine bones, BF: backfat, BL: boneless leg, TL: tenderloin, LB: leg bones, TPB: tail and pelvis bone). The three primal and 14 commercial meat cuts obtained by dissecting the carcass in accordance with the processing standard (GB/T 9959) are called carcass cuts or meat cuts. Details of the carcass cutting process and the appearance of the meat cuts are described in our previous study [9]. Each cut was carefully weighed and measured by our investigators and the MCP were calculated by dividing the weight of the meat cut by the weight of the carcass. Evaluation of the pork loin meat quality followed the same method as outlined in [13, 14]. At 1-d postmortem, seven meat quality traits, namely, pH, L* for lightness (LIL), a* for redness (REA), b* for yellowness (YEB), the proportion of fat areas in the image (PFAI), visual marbling score (VMS) and loin muscle area (LMA), were measured on the *longissimus dorsi* muscle between the 11th rib and the first lumbar vertebra.

### Genotyping

As pedigree information was not available for most individuals, all individuals were genotyped. Genomic DNA was isolated from the muscle tissue of each animal using the routine phenol/chloroform extraction method. The CC1 PorcineSNP50 BeadChip (51,368 SNPs) was used to genotype individuals in accordance with the manufacturer’s protocol. Our previous study [13] provided information on marker density and accuracy of the CC1 PorcineSNP50 BeadChip. All samples had a call rate higher than 90%. SNPs with a call rate lower than 95%, a minor allele frequency (MAF) lower than 5%, or that were in Hardy–Weinberg disequilibrium (P < 10E−5) were filtered out using the PLINK (v1.90b6.24) software [15]. In total, 2012 animals and 40,016 SNPs passed quality control and were included for further statistical analysis.

### Model and estimation of variance components

The following two-trait animal model was used in the analyses:

$$\left[\begin{array}{c}{\mathbf{y}}_{\mathbf{1}}\\ {\mathbf{y}}_{\mathbf{2}}\end{array}\right]=\left[\begin{array}{cc}{\mathbf{W}}_{\mathbf{1}}& 0\\ 0& {\mathbf{W}}_{\mathbf{2}}\end{array}\right]\left[\begin{array}{c}{{\varvec{\upalpha}}}_{\mathbf{1}}\\ {{\varvec{\upalpha}}}_{\mathbf{2}}\end{array}\right]+\left[\begin{array}{cc}{\mathbf{Z}}_{\mathbf{1}}& 0\\ 0& {\mathbf{Z}}_{\mathbf{2}}\end{array}\right]\left[\begin{array}{c}{\mathbf{u}}_{\mathbf{1}}\\ {\mathbf{u}}_{\mathbf{2}}\end{array}\right]+\left[\begin{array}{c}{{\varvec{\upepsilon}}}_{\mathbf{1}}\\ {{\varvec{\upepsilon}}}_{\mathbf{2}}\end{array}\right],$$

where ${\mathbf{y}}_{\mathbf{1}}$ and ${\mathbf{y}}_{\mathbf{2}}$ are the vectors of the phenotypes for the two traits to be analyzed; ${{\varvec{\upalpha}}}_{\mathbf{1}}$ and ${{\varvec{\upalpha}}}_{\mathbf{2}}$ are the vectors of the fixed effects of traits 1 and 2, respectively, including sex, population (LD, YK, LY, and DLY), and slaughter batch (two slaughter batches, containing 1054 and 958 pigs respectively); ${\mathbf{u}}_{\mathbf{1}}$ and ${\mathbf{u}}_{\mathbf{2}}$ are the vectors of the random additive genetic effects, which were assumed to follow the multivariate normal distribution $$\left[\begin{array}{c}{\mathbf{u}}_{\mathbf{1}}\\ {\mathbf{u}}_{\mathbf{2}}\end{array}\right]\sim {\mathrm{MVN}}_{\mathrm{n}}\left(\left[\begin{array}{c}0\\ 0\end{array}\right], \mathbf{G}\left[\begin{array}{cc}{\upsigma }_{\mathrm{a}1}^{2}& {\sigma }_{a12}\\ {\sigma }_{a12}& {\upsigma }_{\mathrm{a}2}^{2}\end{array}\right]\right),$$ where $\mathbf{G}$ is the kinship matrix calculated from the genome-wide SNP genotypes, ${\upsigma }_{\mathrm{a}1}^{2}$ and ${\upsigma }_{\mathrm{a}2}^{2}$ are the additive genetic variance for traits 1 and 2, respectively, and ${\upsigma }_{\mathrm{a}12}$ is the additive genetic covariance; and ${\varvec{\upepsilon}}$_1_ and ${\varvec{\upepsilon}}$_2_ are the vectors of residuals, which were assumed to follow the multivariate normal distribution $$\left[\begin{array}{c}{{\varvec{\upepsilon}}}_{\mathbf{1}}\\ {{\varvec{\upepsilon}}}_{\mathbf{2}}\end{array}\right]\sim {\mathrm{MVN}}_{\mathrm{n}}\left(\left[\begin{array}{c}0\\ 0\end{array}\right], {\mathbf{I}}_{\mathrm{n}}\left[\begin{array}{cc}{\upsigma }_{\mathrm{e}1}^{2}& 0\\ 0& {\upsigma }_{\mathrm{e}2}^{2}\end{array}\right]\right),$$ with ${\mathbf{I}}_{\mathrm{n}}$ being an identity matrix; ${\upsigma }_{\mathrm{e}1}^{2}$ and ${\upsigma }_{\mathrm{e}2}^{2}$ are the variance of the residuals for traits 1 and 2, respectively, and $\mathrm{n}$ is the number of individuals phenotyped; ${\mathbf{W}}_{\mathbf{1}},$ ${\mathbf{W}}_{\mathbf{2}},$ ${\mathbf{Z}}_{\mathbf{1}},$ and ${\mathbf{Z}}_{\mathbf{2}}$ are the corresponding incidence matrices for the effects in ${{\varvec{\upalpha}}}_{\mathbf{1}},$ ${{\varvec{\upalpha}}}_{\mathbf{2}},$ ${\mathbf{u}}_{\mathbf{1}},$ and ${\mathbf{u}}_{\mathbf{2}},$ respectively.

The variance–covariance matrix of $\mathbf{y}$ (the vector of phenotypes for a single-trait) can be expressed as:

$$\mathrm{var}\left(\mathbf{y}\right)=\mathbf{G}{\upsigma }_{\mathrm{a}}^{2}+\mathbf{I}{\upsigma }_{\mathrm{e}}^{2}.$$

The genomic relationship matrix ($\mathbf{G}$) was constructed as in VanRaden [16]:

$$\mathbf{G}=\frac{\mathbf{Z}{\mathbf{Z}}{^{\prime}}}{2\sum {p}_{i}(1-{p}_{i})},$$

where $\mathbf{Z}$ is the matrix of centered SNP genotype dosages, with dimensions equal to the number of individuals and the number of SNPs, and ${p}_{i}$ is the frequency of the reference allele at the $i$-th SNP. These allele frequencies were computed based on the method of Gengler et al. [17].

The heritability was then estimated as ${\mathrm{h}}^{2}={\upsigma }_{\mathrm{a}}^{2}/{\upsigma }_{\mathrm{p}}^{2},$ where ${\upsigma }_{\mathrm{p}}^{2}$ is the phenotypic variance and the sum of ${\upsigma }_{\mathrm{a}}^{2}$ and ${\upsigma }_{\mathrm{e}}^{2}$.

Genetic correlations were calculated as follows:

$${\mathrm{r}}_{\mathrm{a}}=\frac{{\upsigma }_{\mathrm{a}12}}{\sqrt{{\upsigma }_{\mathrm{a}1}^{2}*{\upsigma }_{\mathrm{a}2}^{2}}}.$$

Heritabilities and genetic correlations for all traits were estimated by restricted maximum likelihood (REML) methodology using the GCTA software (version 1.93.1beta) [18]. Estimates of heritability for all traits in the combined population were confirmed using the REML method in the DMUAI module of the DMU software (version 6) [19]. As the sample sizes for the LD and DLY populations were small and the heritability estimates for these populations were very different from those for the YK and LY populations, we only estimated heritabilities for the YK, LY, and combined populations.

### Genome-wide association analyses

Genome-wide association studies (GWAS) were performed using the GEMMA software [20] and the following linear mixed model:

$$\mathbf{y}=\mathbf{W}{\varvec{\upalpha}}+\mathbf{X}{\varvec{\upbeta}}+\mathbf{Z}\mathbf{u}+{\varvec{\upepsilon}},$$

$$\mathbf{u} \sim {\mathrm{MVN}}_{\mathrm{n}}\left(0, {\mathbf{G}\upsigma }_{\mathrm{a}}^{2}\right),$$

$${\varvec{\upepsilon}} \sim {\mathrm{MVN}}_{\mathrm{n}}\left(0, {\mathbf{I}}_{\mathrm{n}}{\upsigma }_{\mathrm{e}}^{2}\right),$$

where $\mathbf{y}$ is the vector of phenotypes; $\mathbf{W}$ is the indicator matrix of the fixed effects, including sex, population, and slaughter batch; ${\varvec{\upalpha}}$ is the vector of the corresponding fixed effects; $\mathbf{X}$ is the genotype indicator matrix; ${\varvec{\upbeta}}$ is the vector of SNP substitution effects; $\mathbf{u}$ is the vector of the random polygenic effects, which was assumed to follow a multivariate normal distribution ${\mathrm{MVN}}_{\mathrm{n}}\left(0, {\mathbf{G}\upsigma }_{\mathrm{a}}^{2}\right)$, where $\mathbf{G}$ is the kinship matrix calculated from the genome-wide SNP genotypes; ${\upsigma }_{\mathrm{a}}^{2}$ is the additive genetic variance; $\mathbf{Z}$ is the corresponding incidence matrix for the effects in $\mathbf{u}$; ${\varvec{\upepsilon}}$ is the vector of residual effects, which was assumed to follows a multivariate normal distribution ${\mathrm{MVN}}_{\mathrm{n}}\left(0, {\mathbf{I}}_{\mathrm{n}}{\upsigma }_{\mathrm{e}}^{2}\right)$, where ${\mathbf{I}}_{\mathrm{n}}$ is an identity matrix; ${\upsigma }_{\mathrm{e}}^{2}$ is the variance of the residual; and $\mathrm{n}$ is the number of individuals phenotyped.

For GWAS, statistical significance was set using the Bonferroni correction method. The genome-wide significance threshold was set at 0.05/N, with 1/N indicating a suggestive significance threshold [21, 22], where N is the number of SNPs (40,016) that passed quality control for GWAS, which resulted in *p* value thresholds of 1.25 × 10^–6^ and 2.50 × 10^–5^, respectively.

## Results

### Phenotypes and their heritabilities

**Fig. 1:**
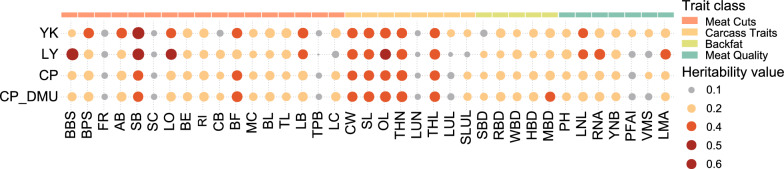
Estimates of heritabilities for all evaluated traits in the four populations. YK, LY and CP represent heritabilities for all traits estimated using the GCTA software in Yorkshire, Landrace Yorkshire and combined populations, respectively. CP_DMU represents heritabilities for all traits estimated using the DMU software in the combined population

Based on our previous results, carcass weight, sex, and breed composition had significant effects on the majority of the meat cut, meat quality, and carcass traits [9]. The coefficient of variation of these traits varied largely from 4.2% for the proportion of LC to 25.3% for the proportion of BF. Here, the heritability was estimated using the GCTA software after adjusting for the effects of carcass weight, sex, and breed composition for the YK, LY, and combined populations (Fig. 1 and Table 1). In addition, we used the DMU software to evaluate the heritability for each trait in the combined population and found that the results were similar to those obtained with the GCTA software (Fig. 1).

**Table 1:** Estimates of heritabilities with their standard error (SE) for carcass cut proportions, carcass traits and meat quality of the* longissimus dorsi* muscle in the LY, YK, and combined (CP) populations

Trait name	Acronym	n	Estimates of heritability ± SE		
			LY	YK	CP
Number of animals evaluated			689	698	2012
Carcass cut proportions					
Primal cuts					
Shoulder cut	SC	1959	0.12 ± 0.06	0.13 ± 0.06	0.14 ± 0.03
Middle cut	MC	1978	0.22 ± 0.07	0.29 ± 0.07	0.27 ± 0.04
Leg cut	LC	1964	0.19 ± 0.07	0.39 ± 0.07	0.31 ± 0.04
Shoulder cuts					
Boneless Boston shoulder	BBS	1964	0.63 ± 0.07	0.23 ± 0.07	0.33 ± 0.04
Boneless picnic shoulder	BPS	1966	0.37 ± 0.07	0.46 ± 0.07	0.39 ± 0.04
Front ribs	FR	1966	0.16 ± 0.07	0.19 ± 0.06	0.15 ± 0.04
Arm bones	AB	1967	0.33 ± 0.08	0.46 ± 0.07	0.38 ± 0.04
Scapula bones	SB	1965	0.59 ± 0.07	0.63 ± 0.07	0.44 ± 0.04
Middle cuts					
Loin	LO	1985	0.52 ± 0.07	0.42 ± 0.07	0.36 ± 0.04
Belly	BE	1993	0.30 ± 0.07	0.36 ± 0.07	0.32 ± 0.04
Ribs	RI	1974	0.31 ± 0.07	0.35 ± 0.07	0.37 ± 0.04
Chine bones	CB	1976	0.33 ± 0.08	0.17 ± 0.06	0.23 ± 0.04
Back fat	BF	1984	0.38 ± 0.08	0.45 ± 0.07	0.44 ± 0.04
Leg cuts					
Boneless leg	BL	1988	0.28 ± 0.08	0.40 ± 0.07	0.34 ± 0.04
Tenderloin	TL	1876	0.33 ± 0.08	0.40 ± 0.07	0.27 ± 0.04
Leg bones	LB	1988	0.40 ± 0.08	0.47 ± 0.07	0.38 ± 0.04
Tail and pelvis bone	TPB	1724	0.03 ± 0.06	0.13 ± 0.06	0.13 ± 0.04
Carcass traits					
Carcass weight	CW	1997	0.49 ± 0.08	0.45 ± 0.07	0.39 ± 0.05
Half carcass weight	HCW	1997	0.46 ± 0.07	0.47 ± 0.07	0.40 ± 0.04
Carcass straight length	CSL	2008	0.45 ± 0.07	0.47 ± 0.07	0.45 ± 0.04
Carcass oblique length	COL	2008	0.50 ± 0.07	0.48 ± 0.07	0.45 ± 0.04
Thoracic length	THL	2011	0.47 ± 0.07	0.44 ± 0.07	0.43 ± 0.04
Lumbar length	LUL	2010	0.07 ± 0.05	0.24 ± 0.07	0.18 ± 0.04
Thoracic number	THN	2012	0.48 ± 0.07	0.45 ± 0.07	0.48 ± 0.04
Lumbar number	LUN	2010	0.09 ± 0.06	0.12 ± 0.06	0.12 ± 0.03
Single lumbar length	SLUL	2010	0.12 ± 0.06	0.23 ± 0.06	0.24 ± 0.04
Backfat depth					
Shoulder backfat depth	SBD	2012	0.31 ± 0.08	0.23 ± 0.06	0.26 ± 0.04
6th_7th rib backfat depth	RBD	2012	0.33 ± 0.08	0.33 ± 0.07	0.32 ± 0.04
Waist backfat depth	WBD	2012	0.32 ± 0.08	0.20 ± 0.06	0.23 ± 0.04
Hip backfat depth	HBD	2012	0.23 ± 0.07	0.25 ± 0.07	0.32 ± 0.04
Mean of backfat depth	MBD	2012	0.39 ± 0.08	0.41 ± 0.07	0.43 ± 0.04
Meat quality					
pH		2010	0.40 ± 0.08	0.29 ± 0.06	0.28 ± 0.04
Lightness, L*	LIL	2000	0.40 ± 0.08	0.40 ± 0.07	0.34 ± 0.04
Redness, a*	REA	2000	0.50 ± 0.07	0.39 ± 0.07	0.34 ± 0.04
Yellowness, b*	YEB	2000	0.35 ± 0.07	0.31 ± 0.07	0.30 ± 0.04
PFAI		1705	0.17 ± 0.08	0.23 ± 0.07	0.20 ± 0.04
Visual marbling score	VMS	1705	0.19 ± 0.08	0.27 ± 0.07	0.20 ± 0.04
Loin muscle area	LMA	1949	0.45 ± 0.07	0.35 ± 0.07	0.29 ± 0.04

*LY* Landrace Yorkshire, *YK* Yorkshire, *CP* combined population, *PFAI* proportion of fat areas in the image

The estimates of heritability (± SE) for MCP ranged from 0.13 ± 0.04 for TPB to 0.44 ± 0.04 for SB in the combined population (Fig. 1 and Table 1). The estimates of heritability for SC, FR, and TPB were low across all populations, and in the combined population they were equal to 0.14 ± 0.03, 0.15 ± 0.04, and 0.13 ± 0.04, respectively. The estimates of heritability for SB, LO, BF, LB, BPS, and BBS in the combined population were 0.44 ± 0.04, 0.36 ± 0.04, 0.44 ± 0.04, 0.38 ± 0.04, 0.39 ± 0.04, and 0.33 ± 0.04, respectively, which are among the highest estimates of heritability found in this study. Overall, the estimates of the heritability for most MCP were moderate to high, which indicates that these traits can be improved in swine breeding programs.

Genetic parameters for carcass traits and meat quality have been investigated for a long time [10–12]. Most studies have reported moderate to high heritability estimates for most carcass traits and low to moderate heritability estimates for meat quality traits. As expected, the heritability estimates for carcass traits ranged from 0.39 ± 0.05 for carcass weight in the combined population to 0.50 ± 0.07 for carcass oblique length in the LY population, and those for meat quality ranged from 0.20 ± 0.04 for VMS to 0.34 ± 0.04 for LIL in the combined population (Fig. 1 and Table 1). Our results confirm the findings of most of the genetic parameter estimates previously reported for carcass and meat quality traits.

### Genetic correlations between MCP, meat quality, and carcass traits

**Fig. 2:**
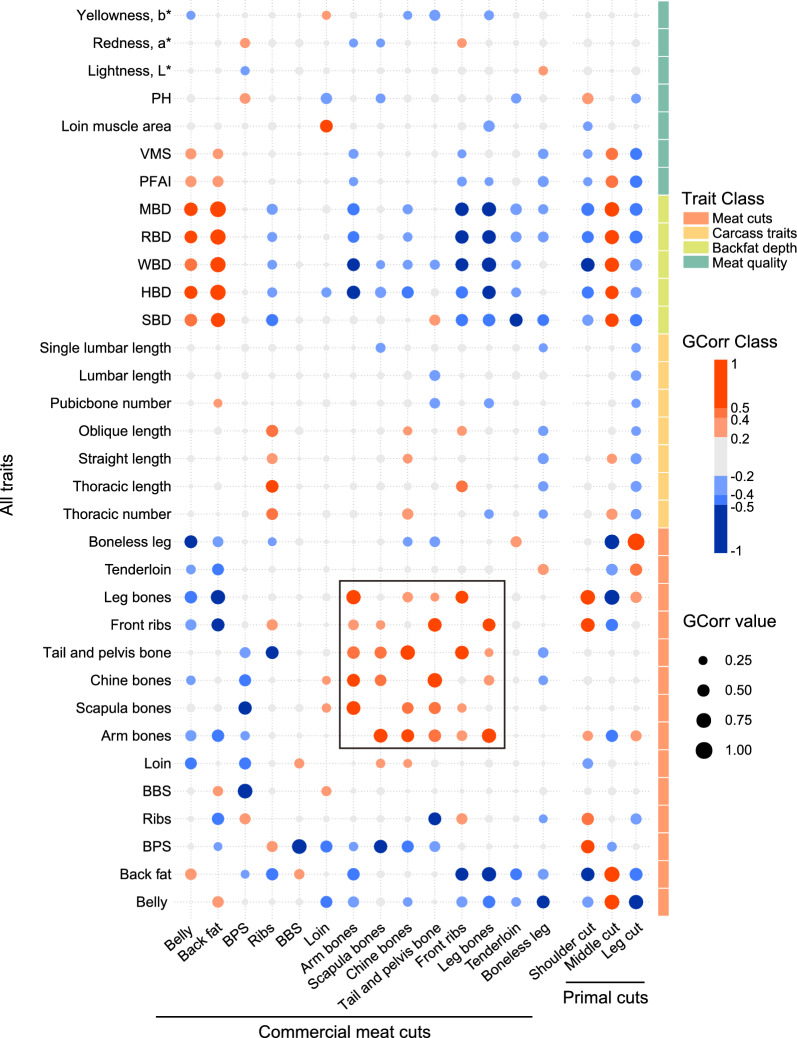
Estimates of genetic correlations of meat cut proportions with carcass and meat quality traits in the combined population

In the combined population, we estimated the genetic correlations between MCP, meat quality, and carcass traits (Fig. 2 and [see Additional file 1: Table S1]). Among these, BF was positively correlated with BE, with a genetic correlation estimate of 0.40 ± 0.08, whereas BF and BE had negative correlation estimates with other meat cuts (except BBS, ranging from -0.26 ± 0.10 between BE and TL to − 0.67 ± 0.06 between BF and LB). In addition, BF and BE were genetically positively correlated with backfat depth (ranging from 0.49 ± 0.09 to 0.84 ± 0.04), PFAI (ranging from 0.36 ± 0.11 to 0.38 ± 0.13), and VMS (ranging from 0.32 ± 0.11 to 0.36 ± 0.12), suggesting that selection to increase the proportion of BE may increase backfat depth and the proportion of BF, which would be detrimental to carcass sales. In addition, BPS was positively genetically correlated with the proportion of RI (0.36 ± 0.08), but negatively correlated with TPB (− 0.34 ± 0.13), CB (− 0.44 ± 0.09), SB (− 0.56 ± 0.07), AB (− 0.24 ± 0.09), LO (− 0.44 ± 0.08), BBS (− 0.71 ± 0.07), and BF (− 0.20 ± 0.08) [Fig. 2 and (see Additional file 1: Table S1)]. Rib proportion was moderately to highly positively genetically correlated with COL (0.45 ± 0.07), CSL (0.35 ± 0.08), THL (0.50 ± 0.07), and THN (0.42 ± 0.08), and negatively correlated with backfat depth (ranging from − 0.26 ± 0.10 to − 0.45 ± 0.10). The proportion of LO was highly positively genetically correlated with LMA (0.51 ± 0.08) but negatively correlated with pH (− 0.34 ± 0.13). Moreover, TL was positively correlated with BL, with a genetic correlation estimate of 0.36 ± 0.10, but was negatively correlated with backfat depth (ranging from − 0.24 ± 0.11 between TL and WBD to − 0.53 ± 0.1 between TL and SBD). However, BL was genetically slightly unfavorably correlated with carcass traits (ranging from − 0.21 ± 0.11 to − 0.35 ± 0.08).

It is worth noting that positive genetic correlations were estimated among the bone carcass cuts of LB, FR, TPB, CB, SB, and AB (ranging from 0.20 ± 0.13 between TPB and LB to 0.69 ± 0.14 between TPB and CB) but negative genetic correlations were estimated for these traits with BF, BE, BPS, and BL [Fig. 2 and (see Additional file 1: Table S1)]. These bone carcass cuts were also estimated to be negatively genetically correlated with backfat depth (ranging from − 0.21 ± 0.09 between SB and WBD to − 0.66 ± 0.06 between LB and MBD), except for the positive genetic correlation estimate between TPB and SBD (0.35 ± 0.16). The results indicate that, in the pig, the growth and development of bones in different parts of the body are synchronized.

As shown in Fig. 2 and Additional file 1: Table S1, we found that the genetic correlations between most meat cut traits were low (correlations lower than 0.3) or not significant (*p* value > 0.05). In addition, the majority of the meat cut traits had low or nonsignificant genetic correlations with carcass and meat quality traits. This is consistent with previously reported results [9] of low or nonsignificant phenotypic correlations between meat cut, carcass, and meat quality traits (Fig. 2).

### Genome-wide association analysis of carcass cut traits

Based on 40,016 SNPs and 2012 individuals, we identified 28 QTL that were associated with 15 carcass cut traits and reached the suggestive significance threshold (Table 2). There was no population stratification, as shown in the Q-Q plots in Additional file 2: Fig. S1. Among these QTL, six had a top SNP that exceeded the genome-wide significance threshold (*P* = 1.25 × 10^–6^).

**Table 2:** Single nucleotide polymorphisms that are significantly associated with carcass cut proportions detected in the single-trait genome-wide association studies

Traits	TOP	Chr	Pos (bp)	*p* value	Nearest gene	Dis (bp)	Candidate gene
BBS	rs1501030	15	44,344,760	1.72E−05	*TENM3*	Within	
BPS	rs0702610	7	97,615,897	9.33E−06	*VRTN*	Within	
AB	rs0700767	7	30,100,555	1.37E−08	*GRM4*	78,750	*HMGA1*, *NUDT3, GRM4*
AB	rs0501169	5	66,142,277	2.19E−06	*CCND2*	27,706	
AB	rs1705053	17	15,896,846	2.25E−05	*BMP2*	135,651	*HAO1, BMP2*
SB	rs1501163	15	50,915,343	5.05E−06	*UNC5D*	112,675	
SC	rs0702610	7	97,615,897	2.76E−06	*VRTN*	Within	
SC	rs0702516	7	121,223,217	2.01E−05	*WARS1*	Within	*DLK1*
SC	rs0201138	2	60,064,913	1.19E−05	*MAP1S*	35,048	
LO	rs0501038	5	57,616,938	9.18E−06	*ERP27*	Within	
BE	rs1806217	18	10,455,423	1.20E−05	*UBN2*	4168	
BE	rs0200123	2	6,851,449	2.45E−05	*SLC25A45*	168	
RI	rs0702610	7	97,615,897	4.18E−16	*VRTN*	Within	*VRTN*
CB	rs0702038	7	97,618,073	6.94E−08	*VRTN*	Within	*VRTN*
CB	rs1402627	14	125,929,866	2.77E−06	*ATRNL1*	Within	
CB	rs1500252	15	9,945,936	1.24E−05	*LRP1B*	Within	
CB	rs1705053	17	15,896,846	1.53E−05	*BMP2*	135,651	*BMP2*
BF	rs1100173	11	9,429,262	8.14E−06	*KL*	Within	
MC	rs0702610	7	97,615,897	4.47E−13	*VRTN*	Within	
BL	rs1704784	17	2,382,922	7.70E−06	*SGCZ*	Within	
TL	rs1303072	13	148,117,239	1.08E−05	*CD96*	Within	
TL	rs0602330	6	123,281,566	2.04E−05	-	-	
LB	rs1705079	17	16,788,523	2.67E−14	*HAO1*	Within	*BMP2, BMP2*
LB	rs0501167	5	66,103,958	5.84E−08	*CCND2*	Within	
LB	rs0700777	7	30,235,367	3.80E−06	*GRM4*	Within	*HMGA1*, *NUDT3, GRM4*
LB	rs1300094	13	3,477,035	1.02E−05	*RFTN1*	Within	
LC	rs1603764	16	32,867,067	1.58E−05	*NDUFS4*	7383	
LC	rs1806864	18	42,102,045	2.00E−05	*MINDY4*	Within	

**Fig. 3:**
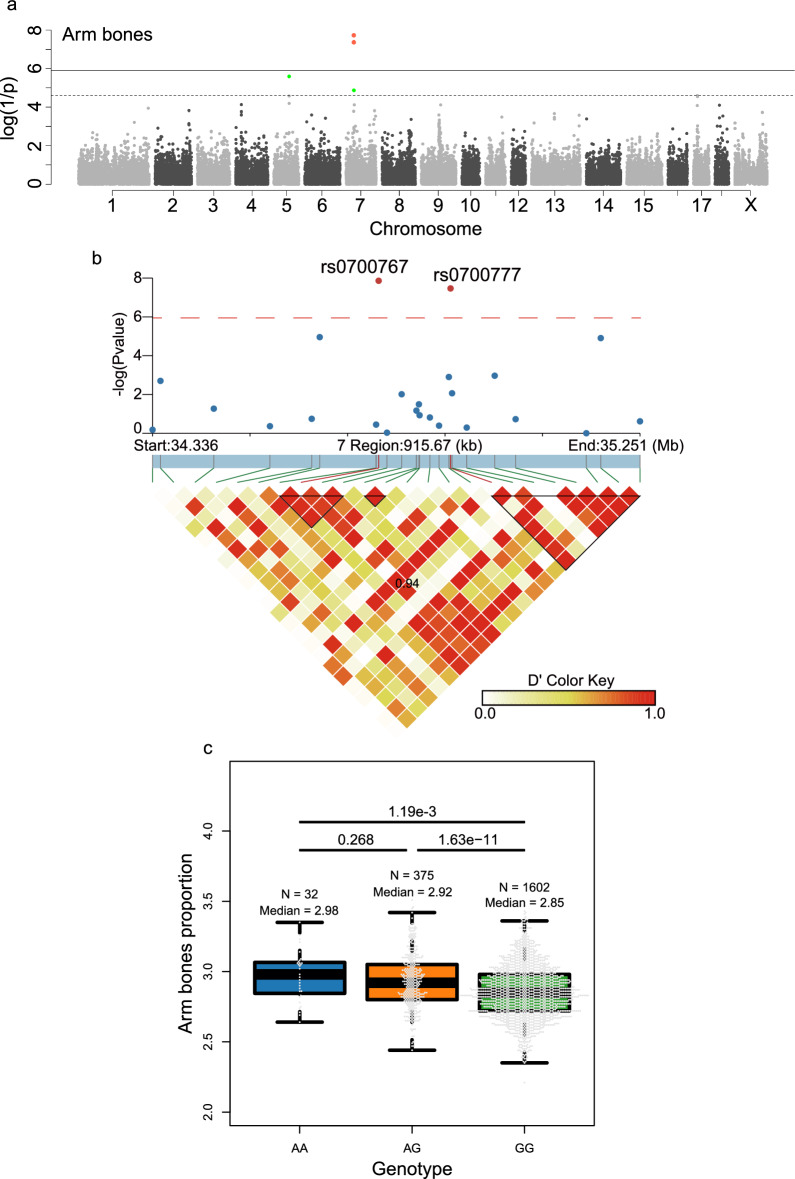
Genome-wide association study for the proportion of arm bones. **a** The Manhattan plot shows the associations of 40,016 SNPs with proportion of arm bones in the combined population. The red dots represent SNPs that reach the genome-wide significance threshold (P = 1.25 × 10–6). **b** A haplotype view of all polymorphic sites within the 500-kb interval before and after the top significant SNP. **c** Box plots showing the differences in proportion of arm bones according to genotype

For shoulder cut traits, two genome-wide significant SNPs and 20 suggestive significant (2.50 × 10^–5^) SNPs were identified to be associated with the proportion of BBS, BPS, AB and SC. Among the SNPs in the region between 29.99 and 30.57 Mb on the *Sus scrofa* chromosome (SSC) 7, two genome-wide significant SNPs were identified to be associated with the proportion of AB (Fig. 3a). The most significant SNP associated with the proportion of AB was located at position 30,100,555 bp on SSC7 (rs0700767) with a *p* value of 1.37 × 10^–8^, and was in strong linkage disequilibrium (D' = 0.94) with SNP rs0700777 (Fig. 3b). Phenotypes were corrected using the linear mixed model for GWAS, and the phenotypes of individuals were grouped into three classes according to their genotypes (*AA*, *AG*, and *GG*) at this locus (30,100,555 bp). Student’s t-test was performed between each pair of the three classes (Fig. 3d). The pairwise t-test *p* value were 0.268, 1.19 × 10^–3^, and 1.63 × 10^–11^ for *AA* vs. *AG*, *AA* vs. *GG*, and *AG* vs. *GG*, respectively (Fig. 3d). Although the difference between individuals with genotypes *AA* and *AG* was nonsignificant, this might be due to a smaller number of individuals with the *AA* genotype. Notably, the 29.99–30.57 Mb region on SSC7 includes four genes that have been previously reported as potential candidates for limb bone length [23] and body height in pigs, namely *GRM4, HMGA1, RPS10* and *NUDT3* [24, 25], and also with body mass index and height in humans [26, 27].

**Fig. 4:**
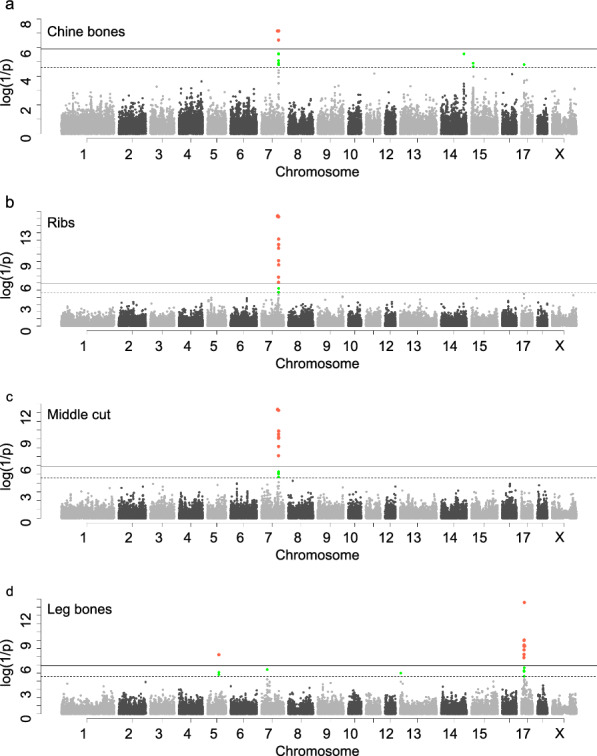
Genome-wide association study for proportion of chine bones, ribs, middle cut, and leg bones. **a**–**d** The Manhattan plot shows the associations of 40,016 SNPs with proportion of chine bones, ribs, middle cut and leg bones, respectively, in the combined population

For middle cut traits, multiple QTL in a 6.11-Mb region (from 97.61 to 103.72 Mb) on SSC7 that included 12, 4, and 10 genome-wide significant SNPs were identified to be associated with RI, CB, and MC proportions, respectively (Table 2). A previous study identified a causal mutation (g.19034 *A* > *C*) in the *VRTN* gene located within this region that affects thoracic vertebrae number and performed a series of functional validation experiments [28]. The number of ribs and the THL change with THN and we also identified QTL that were significantly associated with THN and THL in the same genomic region (see Additional file 3: Fig. S2a, b). The most significant SNP for THN was located at position 97,614,602 bp (rs0702609), with a *p* value of 2.02 × 10^–107^, and this locus corresponded exactly to the causal mutation g.19034 *A* > *C*. The most significant SNP for THL (*P* = 1.77 × 10^–46^), located within the *VRTN* gene at position 97,618,073 bp (rs0702038), was also the most significant SNP for CB (*P* = 6.94 × 10^–8^, Fig. 4a), and was in complete linkage disequilibrium with rs0702689 (see Additional file 3: Fig. S2c). The most significant SNP for both RI and MC proportions was rs0702610 (*P*_RI_ = 4.18 × 10^–16^,* P*_MC_ = 4.47 × 10^–13^, Fig. 4b, c, located within the *VRTN* gene at position 97,615,897 bp, which is in complete linkage disequilibrium with rs0702689 and rs0702609 (see Additional file 3: Fig. S2c). This suggests that alterations in THN and rib number due to causal variants within the *VRTN* gene can influence RI, CB and MC proportions.

For leg cut traits, we identified nine genome-wide significant SNPs and 14 suggestive significant SNPs that are associated with the proportion of BL, TL, LB, and LC (Table 2 and Fig. 4d). Specifically, one SNP on SSC5 and eight SNPs on SSC17 were significantly (*P* < 1.25 × 10^–6^) associated with LB proportion (Fig. 4d). The region containing the eight significant SNPs on SSC17 covers the *BMP2* gene, a member of the bone morphogenetic protein (BMP) family, which is a candidate gene for LB proportion. Abnormal expression of *BMP2* results in failure of some chondrogenic condensates to form in the limbs, which affects vertebrate limb development and skeletogenesis [29, 30]. The most significant SNP for LBP was located at position 16,788,523 bp on SSC17 (rs0700767), with a *p* value of 2.67 × 10^–14^, explaining 2.7% of the phenotypic variation. The other top SNP associated with LBP on SSC5 was located at position 66,103,958 bp (rs0501167) with a *p* value of 5.84 × 10^–8^, and was within the *CCND2* gene. In previous studies, the *CCND2* gene has been shown to regulate the cell cycle progression of hematopoietic stem cells [31], and SNPs in *CCND2* have been found to be significantly associated with human height [32]. Moreover, An et al*.* [33] identified *CCND2* as a candidate gene for hip height, body height, and body length in Chinese Wagyu beef cattle, while Le et al*.* [34] reported that *CCND2* participates in growth-related processes and regulates conformational traits in pigs. These results suggest that *BMP2* and *CCND2* are candidate genes for LB.

## Discussion

Cutting carcasses into different types of cut products can efficiently increase their economic value. As a result, an increasing number of academics and breeding companies are interested in selecting for carcass traits. Scholars have investigated the process of carcass cuts and yield segmentation production since the 1970s [5, 6]. Recent studies [7, 8], including our previous study [9], have investigated the effects of sex, breed, carcass weight, and other factors on meat cut traits. Using pedigree information, Newcom et al. [35], van Wijk et al. [12], and Miar et al. [11] estimated the heritability of such traits as well as their genetic correlations with other carcass features and with meat quality traits. They found that the heritability for the majority of meat cut traits is moderate and that the genetic correlations between these traits are low. Here, we constructed a kinship matrix using the genotypes for 40,016 SNPs and evaluated the genetic parameters of meat cut traits in various breeds. Our results are comparable to those of previous studies. In addition, based on the GB/T 9959 carcass cutting standards, we evaluated the genetic parameters of other meat cut traits than those that were evaluated in most previous reports, including AB, SB, CB, LB, and TPB cut proportions, which are favored by Chinese consumers.

The study of the heritability of meat cut traits from different breeds revealed that most of them had moderate to high heritabilities, which suggests that there is great potential to improve these traits in breeding programs. However, when planning breeding for meat cut traits, the genetic correlations of such traits of interest with other economically important traits must also be considered. Although the middle cut has a higher retail value than other primal cuts and the proportion of MC has a favorable positive genetic correlation with both BE proportion and intramuscular fat content, it also has a positive genetic correlation with BF proportion and backfat depth. Therefore, breeding to improve the proportion of MC may lead to an undesirable increase in backfat. In addition, rib proportion, which has the greatest retail value, was favorably positively correlated with carcass oblique length, straight length, thoracic number, and thoracic length and was also negatively correlated with backfat. Loin, which is a popular meat cut among consumers, shows a favorable genetic positive correlation with loin muscle area and a negative correlation with backfat depth. Selection for these two cut traits would also benefit fatness traits, which is consistent with results of previous studies [10–12]. In general, the majority of meat cuts had low or nonsignificant genetic correlations with carcass and meat quality traits, which indicates that breeding selection for carcass cuts has a limited impact on carcass and meat quality traits.

Meat cuts are post-slaughter carcass composition traits that are challenging to evaluate in vivo. Therefore, marker-assisted selection and genomic selection would be optimal methods to select for carcass cuts, as these strategies can shorten the generation interval through early selection and increase the accuracy of the predicted breeding values, especially for complex traits with a low heritability and traits that are difficult to measure [36–39]. However, few studies have dissected the genetic mechanisms that underlie meat cut traits. In this study, we report the detection of 28 QTL that are significantly associated with meat cuts through GWAS. For example, the causal SNP (g.19034 *A* > *C*) in the *VRTN* gene that is associated with thoracic vertebrae number [28] is also significantly associated with the proportions of middle cut, chine bones, ribs, shoulder cut and picnic shoulder. In addition, in previous reports, several candidate genes located near these QTL that are significantly associated with the proportions of arm bone and leg bone such as *BMP2*, *GRM4*, *HMGA1*, *CCND2*, *HAO1,* and *NUDT3*, have all been found to be associated with skeletal development [26, 27, 29, 30, 32] and height in humans [26, 27, 32], and with hip height, body height, and body length of pigs [23, 25, 34]. Overall, the QTL and candidate genes identified here for meat cut traits are relevant for the selective breeding for meat cut traits and provide an essential basis for further research of the genetic mechanism underlying meat cut traits.

The body shape of pigs includes two dimensions: body length (spine) and body height (forelimbs and hindlimbs). The carcass cuts in this study were divided based on the pig’s bone structure and commercial value, including the three types of bones: arm bones (forelimb), chine bones (spine), and leg bones (hindlimbs). We identified three candidate genes that are significantly associated with these three types of skeletal development, namely *HMGA1*, *VRTN,* and *BMP2* [26–30, 32]. Among these, *HMGA1* may be the strongest candidate gene for forelimb bone development, *VRTN* is a causal gene for vertebra number, as we have previously shown, and *BMP2* may be the strongest candidate gene for hindlimb bone development. Therefore, it seems that bone development in different parts of the body is regulated by different genes. Analysis of the genetic architecture of meat cut traits will help analyze the molecular mechanism that underlies bone development of the forelimbs, spine and hindlimbs and lays a research foundation for the selection of the best shapes in different parts of the body.

## Conclusions

This study evaluated the genetic parameters of 17 carcass cut traits, 12 carcass traits, and six meat quality traits in four populations of pigs. Genetic parameters for some of these novel meat cut traits have not been reported before. We found that some high economic value meat cuts, such as ribs, belly, loin, and tenderloin, have moderate to high heritabilities, which indicates that improvement of these traits through breeding programs is possible. This could increase the market price of the carcass by selecting on key meat cut traits. Estimates of genetic correlations revealed weak or nonsignificant correlations between many meat cut, carcass, and meat quality traits. Rib proportion was favorably correlated with carcass oblique length, straight length, thoracic number, and thoracic length, and negatively correlated with backfat depth. In addition, the GWAS identified 28 QTL and several candidate genes for meat cut traits for the first time. These findings provide a key reference for marker-assisted selection and genomic selection for meat cut traits.

## Supplementary Information

**Additional file 1: Table S1.** Estimates of genetic correlations between carcass cut proportions with carcass traits and meat quality traits.

**Additional file 2:** **Figure S1.** Q-Q plot and lambda values of genome-wide association study (GWAS) of meat cut traits and carcass traits. Lambda values are normally used to characterize population stratification in multibreed GWAS.

**Additional file 3: Figure S2.** Genome-wide association study (GWAS) for thoracic number and thoracic length. **a**, **b** The Manhattan plots show the associations of 40,016 SNPs with thoracic number and thoracic length, respectively, in the combined population. **c** A haplotype view of all polymorphic sites within the 500-kb interval before and after the top significant SNP (rs0702038).

## Data Availability

The data that support the findings of this study are available from the corresponding author upon reasonable request.

## References

[1] Gong SL, Yang YS, Shen H, Wang XY, Guo HP, Bai L (2011). Meat handling practices in households of mainland china. Food Control.

[2] Liu H, Sun D (2010). China pork consumption situation and outlook. Agric Outlook.

[3] Grunert KG (2006). Future trends and consumer lifestyles with regard to meat consumption. Meat Sci.

[4] Qin J (2019). Evaluation of the value gain on pork economical carcass cutting and its related breeding technology development.

[5] Mandigo RW, Thompson TL, Weiss GM (1979). Commercial accelerated pork processing: yields of fresh carcass cuts. J Food Sci.

[6] Mandigo RW, Thompson TL, Weiss GM (1977). Commercial accelerated pork processing: yields of cured ham, bacon and loins. J Food Sci.

[7] Overholt MF, Arkfeld EK, Mohrhauser DA, King DA, Wheeler TL, Dilger AC (2016). Comparison of variability in pork carcass composition and quality between barrows and gilts. J Anim Sci.

[8] Alvarez-Rodríguez J, Teixeira A (2019). Slaughter weight rather than sex affects carcass cuts and tissue composition of bisaro pigs. Meat Sci.

[9] Xie L, Qin J, Rao L, Cui D, Tang X, Xiao S (2023). Effects of carcass weight, sex and breed composition on meat cuts and carcass trait in finishing pigs. J Integr Agr.

[10] Khanal P, Maltecca C, Schwab C, Gray K, Tiezzi F (2019). Genetic parameters of meat quality, carcass composition, and growth traits in commercial swine. J Anim Sci.

[11] Miar Y, Plastow GS, Moore SS, Manafiazar G, Charagu P, Kemp RA (2014). Genetic and phenotypic parameters for carcass and meat quality traits in commercial crossbred pigs. J Anim Sci.

[12] van Wijk HJ, Arts DJG, Matthews JO, Webster M, Ducro BJ, Knol EF (2005). Genetic parameters for carcass composition and pork quality estimated in a commercial production chain. J Anim Sci.

[13] Xie L, Qin J, Rao L, Tang X, Cui D, Chen L (2021). Accurate prediction and genome-wide association analysis of digital intramuscular fat content in longissimus muscle of pigs. Anim Genet.

[14] Zheng M, Huang Y, Ji J, Xiao S, Ma J, Huang L (2018). Effects of breeds, tissues and genders on purine contents in pork and the relationships between purine content and other meat quality traits. Meat Sci.

[15] Purcell S, Neale B, Todd-Brown K, Thomas L, Ferreira MA, Bender D (2007). Plink: a tool set for whole-genome association and population-based linkage analyses. Am J Hum Genet.

[16] Vanraden PM (2008). Efficient methods to compute genomic predictions. J Dairy Sci.

[17] Gengler N, Mayeres P, Szydlowski M (2007). A simple method to approximate gene content in large pedigree populations: application to the myostatin gene in dual-purpose belgian blue cattle. Animal.

[18] Yang J, Benyamin B, McEvoy BP, Gordon S, Henders AK, Nyholt DR (2010). Common snps explain a large proportion of the heritability for human height. Nat Genet.

[19] Madsen P, Jensen J (2013). A user's guide to DMU. A package for analysing multivariate mixed models. Version 6, release 5.2.

[20] Zhou X, Stephens M (2012). Genome-wide efficient mixed-model analysis for association studies. Nat Genet.

[21] Guo Y, Huang Y, Hou L, Ma J, Chen C, Ai H (2017). Genome-wide detection of genetic markers associated with growth and fatness in four pig populations using four approaches. Genet Sel Evol.

[22] Liu X, Xiong X, Yang J, Zhou L, Yang B, Ai H (2015). Genome-wide association analyses for meat quality traits in chinese erhualian pigs and a western Duroc × (Landrace × Yorkshire) commercial population. Genet Sel Evol.

[23] Zhang L, Li N, Liu X, Liang J, Yan H, Zhao K (2014). A genome-wide association study of limb bone length using a large white × minzhu intercross population. Genet Sel Evol.

[24] Xu J, Fu Y, Hu Y, Yin L, Tang Z, Yin D (2020). Whole genome variants across 57 pig breeds enable comprehensive identification of genetic signatures that underlie breed features. J Anim Sci Biotechnol.

[25] Wang L, Zhang L, Yan H, Liu X, Li N, Liang J (2014). Genome-wide association studies identify the loci for 5 exterior traits in a Large White × Minzhu pig population. PLoS One.

[26] Soranzo N, Rivadeneira F, Chinappen-Horsley U, Malkina I, Richards JB, Hammond N (2009). Meta-analysis of genome-wide scans for human adult stature identifies novel loci and associations with measures of skeletal frame size. PLoS Genet.

[27] Cho YS, Go MJ, Kim YJ, Heo JY, Oh JH, Ban H (2009). A large-scale genome-wide association study of asian populations uncovers genetic factors influencing eight quantitative traits. Nat Genet.

[28] Duan Y, Zhang H, Zhang Z, Gao J, Yang J, Wu Z (2018). VRTN is required for the development of thoracic vertebrae in mammals. Int J Biol Sci.

[29] Rosen V (2009). BMP2 signaling in bone development and repair. Cytokine Growth Factor Rev.

[30] Bandyopadhyay A, Tsuji K, Cox K, Harfe BD, Rosen V, Tabin CJ (2006). Genetic analysis of the roles of BMP2, BMP4, and BMP7 in limb patterning and skeletogenesis. PLoS Genet.

[31] Kamatani Y, Matsuda K, Okada Y, Kubo M, Hosono N, Daigo Y (2010). Genome-wide association study of hematological and biochemical traits in a japanese population. Nat Genet.

[32] Sakaue S, Kanai M, Tanigawa Y, Karjalainen J, Kurki M, Koshiba S (2021). A cross-population atlas of genetic associations for 220 human phenotypes. Nat Genet.

[33] An B, Xia J, Chang T, Wang X, Xu L, Zhang L (2019). Genome-wide association study reveals candidate genes associated with body measurement traits in chinese wagyu beef cattle. Anim Genet.

[34] Le TH, Christensen OF, Nielsen B, Sahana G (2017). Genome-wide association study for conformation traits in three danish pig breeds. Genet Sel Evol.

[35] Newcom DW, Baas TJ, Mabry JW, Goodwin RN (2002). Genetic parameters for pork carcass components. J Anim Sci.

[36] Meuwissen TH, Hayes BJ, Goddard ME (2001). Prediction of total genetic value using genome-wide dense marker maps. Genetics.

[37] García-Ruiz A, Cole JB, Vanraden PM, Wiggans GR, Ruiz-López FJ, Van Tassell CP (2016). Changes in genetic selection differentials and generation intervals in us holstein dairy cattle as a result of genomic selection. Proc Natl Acad Sci USA.

[38] Nejati-Javaremi A, Smith C, Gibson JP (1997). Effect of total allelic relationship on accuracy of evaluation and response to selection. J Anim Sci.

[39] Meuwissen T, Goddard M (2010). Accurate prediction of genetic values for complex traits by whole-genome resequencing. Genetics.

