# Supplementary figures and images for: Genetic dissection of 26 meat cut, meat quality and carcass traits in four pig populations
Source: Genet Sel Evol. 2023 Jun 29;55:43. doi: 10.1186/s12711-023-00817-y (PMC10311868; doi:10.1186/s12711-023-00817-y)

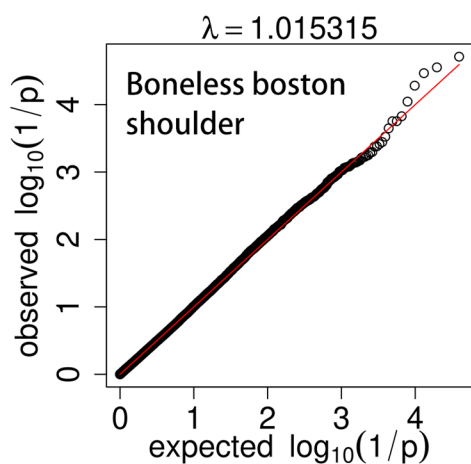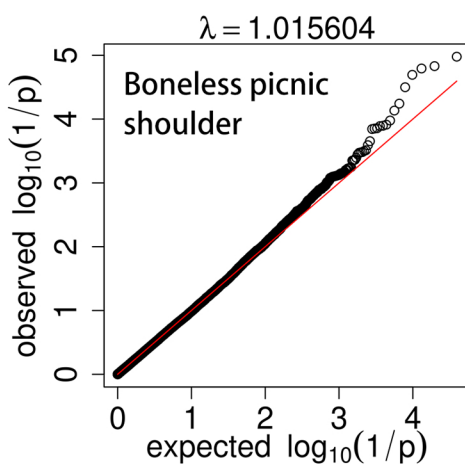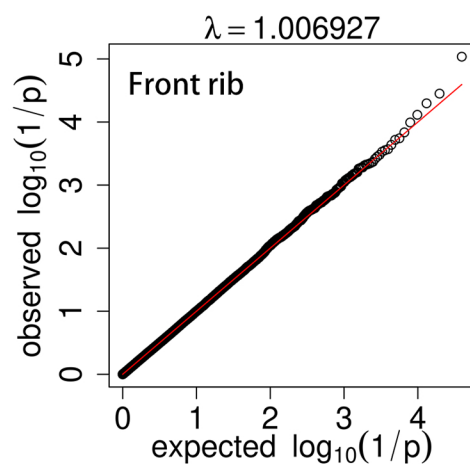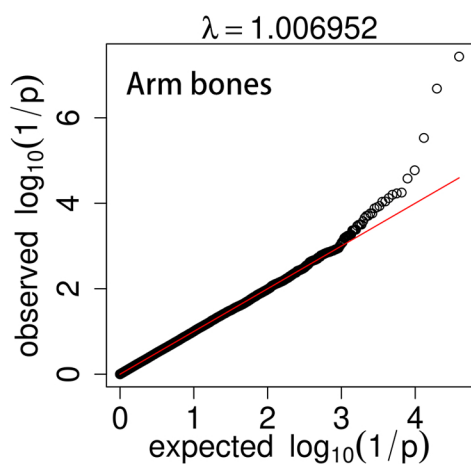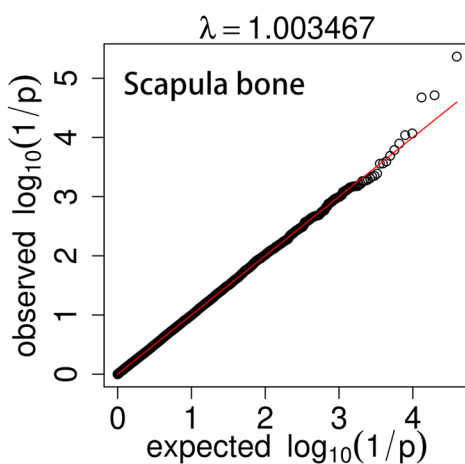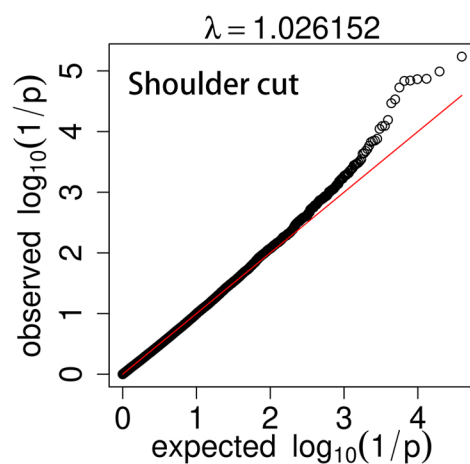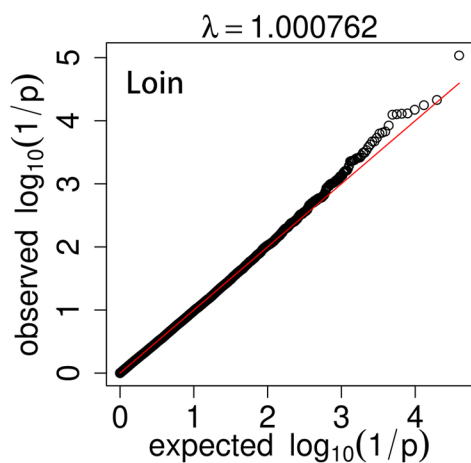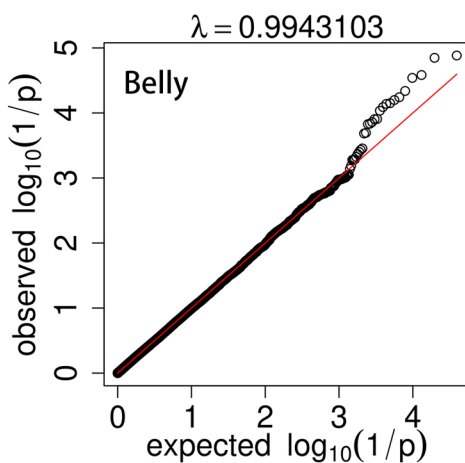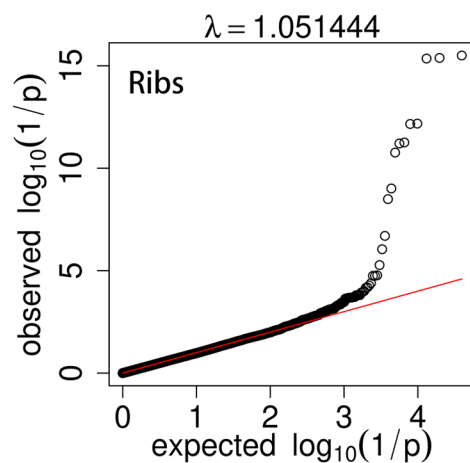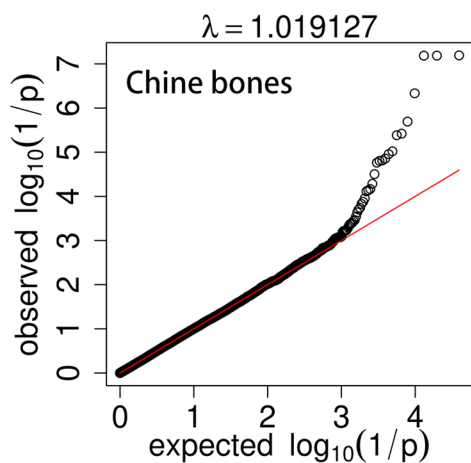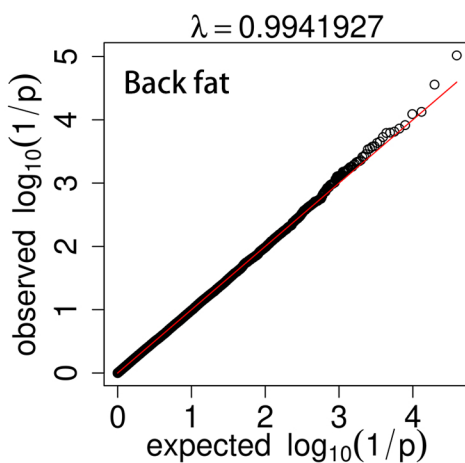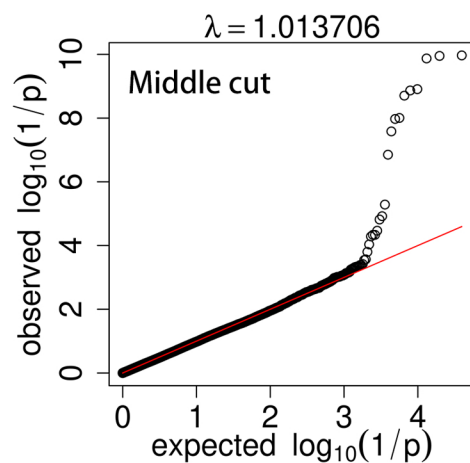

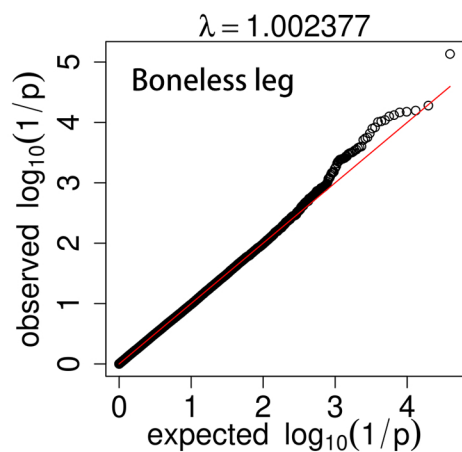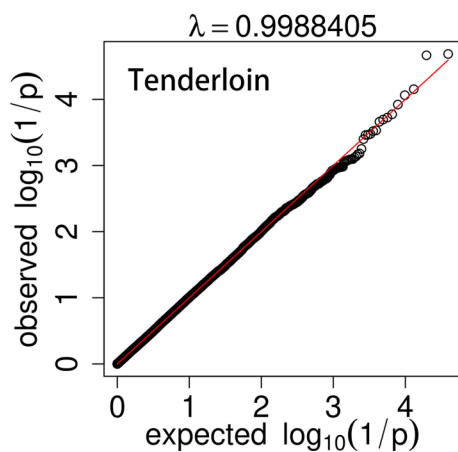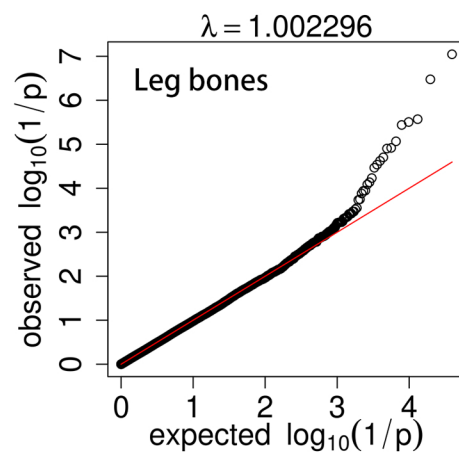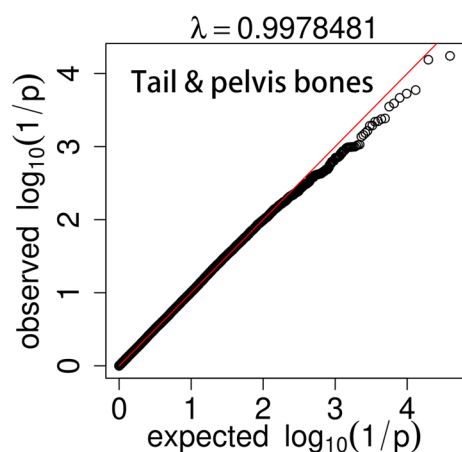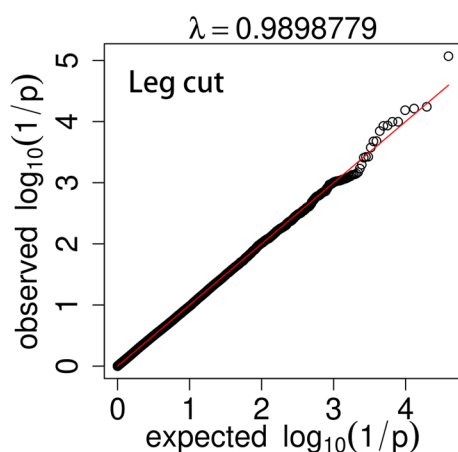

Supplement: Supplementary file 2 — Additional file 2: Figure S1. Q-Q plot and lambda values of genome-wide association study (GWAS) of meat cut traits and carcass traits. Lambda values are normally used to characterize population stratification in multibreed GWAS. [file 12711_2023_817_MOESM2_ESM.pdf]

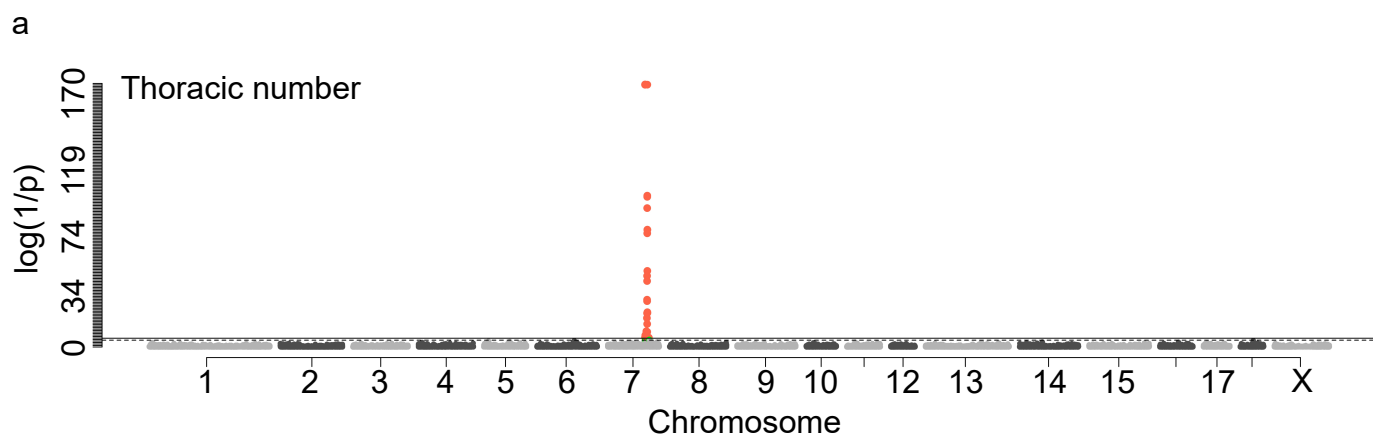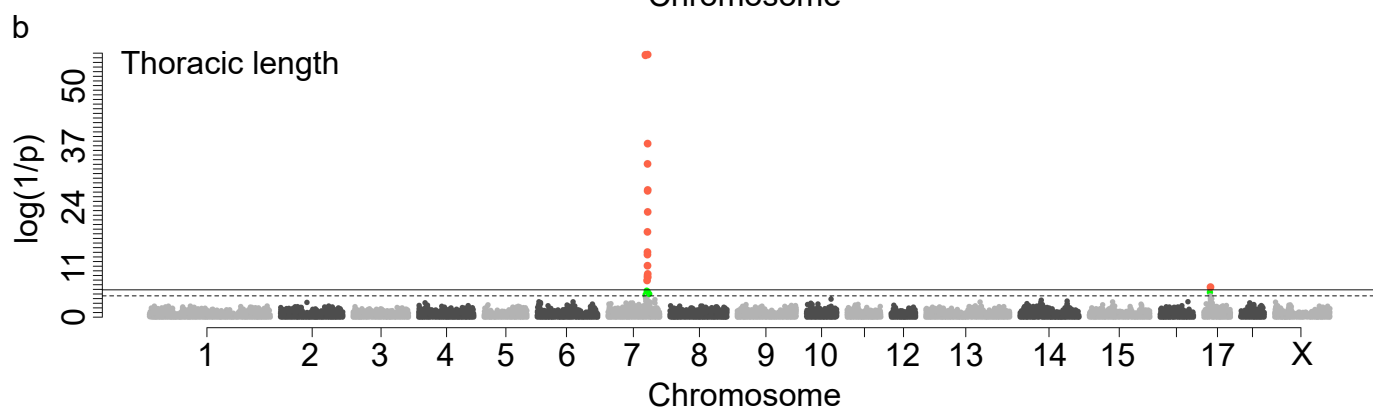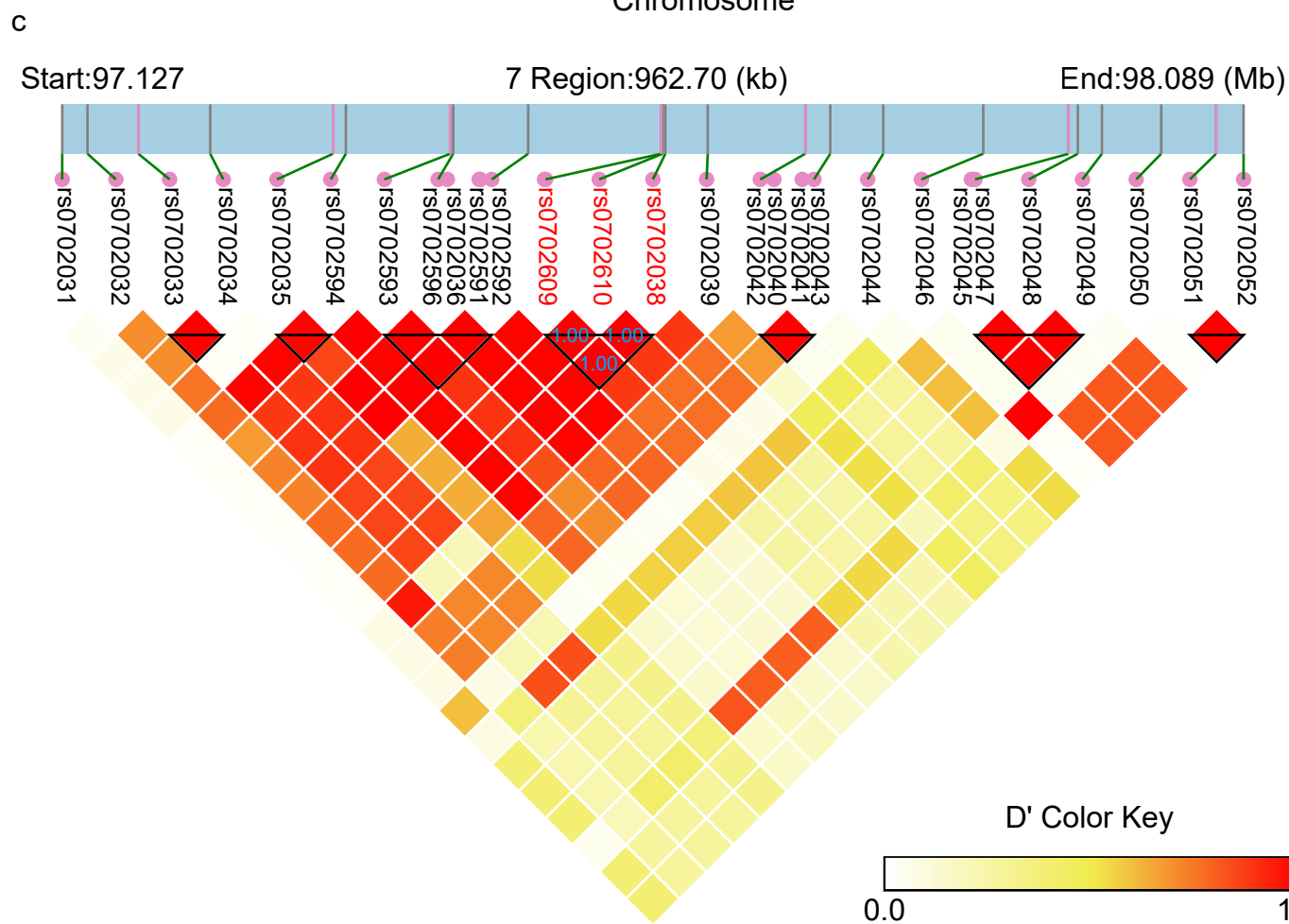

Supplement: Supplementary file 3 — Additional file 3: Figure S2. Genome-wide association study (GWAS) for thoracic number and thoracic length. a, b The Manhattan plots show the associations of 40,016 SNPs with thoracic number and thoracic length, respectively, in the combined population. c A haplotype view of all polymorphic sites within the 500-kb interval before and after the top significant SNP (rs0702038). [file 12711_2023_817_MOESM3_ESM.pdf]
